# Impact of Post-Exodontia Bleeding in Cardiovascular Patients: A New Classification Proposal

**DOI:** 10.2174/1874192401711010102

**Published:** 2017-09-30

**Authors:** T. Lillis, M. Didagelos, L. Lillis, C. Theodoridis, H. Karvounis, A. Ziakas

**Affiliations:** 1 Department of Oral Surgery, Implantology and Radiology, School of Dentistry, Faculty of Health Sciences, Aristotle University of Thessaloniki, Thessaloniki, Greece; 2 1^st^ Department of Cardiology, Aristotle University of Thessaloniki, AHEPA University Hospital, Thessaloniki, Thessaloniki, Greece

**Keywords:** Dental extraction, Bleeding, Classification, Antiplatelet, Anticoagulant

## Abstract

**Background:**

Exodontia (dental extraction), being the most frequent minor surgical procedure in the general population, inevitably involves a large number of patients on antithrombotic medication. Current experience shows that there is a degree of confusion in managing these patients.

**Description:**

Post-exodontia bleeding, a natural consequence of every dental extraction with no or minor clinical significance in the vast majority of cases, often appears to be of major concern to both patients and healthcare practitioners (dentists or physicians), either because of the alarming nature of oral bleeding itself or because of the distorted perception about its importance. These concerns are enhanced by the lack of a universal standardized definition of post-exodontia bleeding and by the fact that all currently available post-exodontia bleeding definitions bear intrinsic limitations and tend to overestimate its clinical significance.

**Conclusion:**

In order to overcome the aforementioned issues, this article presents an overview of post-extraction bleeding and proposes a classification, based on the well-recognized Bleeding Academic Research Consortium (BARC) bleeding definition, aiming at reducing heterogeneity in this field.

## 1 INTRODUCTION

Exodontia (dental extraction) is a frequent minor surgical procedure in the general population. Post-exodontia bleeding is a common problem for many patients and practitioners. Although documented, life-threatening post-exodontia bleeding complications are rare [1]. However, intra-oral bleeding and bloody taste are alarming signs for the majority of people, leading them to seek help from a healthcare professional.

The issue becomes more complicated given that there is a growing population of patients receiving long-term antithrombotic therapy due to the rising prevalence of cardiovascular diseases, but also due to expanding indications of antithrombotic therapy with the increasing use of invasive cardiac procedures [1]. In these patients the risk of potential bleeding complications related to dental surgery should be weighed against the risk of embolic complications if antithrombotic medication is temporarily withdrawn. Despite the increasing evidence from clinical studies that high morbidity bleeding complications are uncommon when patients on antithrombotics undergo dental surgery, surveys have shown that dentists and physicians usually overestimate the risk of postoperative bleeding and thus prefer to interrupt antithrombotic medication perioperatively [2-6].

The primary aim of the present analysis is the comprehensive review of bleeding complications related to exodontia, in order to provide a useful aid for dental and medical practitioners in clinical decision making. The secondary aim is the proposal of a classification for bleeding complications related to exodontia in order to help researchers correctly classify bleeding events when conducting antithrombotic clinical trials.

## 2 POST-EXODONTIA BLEEDING OVERVIEW

The time course of post-exodontia bleeding begins with the immediate post-extraction bleeding which can be defined as the primary haemorrhage coming from the freshly damaged capillaries of the socket (gingivae, periodontal ligament and bony walls) immediately after tooth removal. Brennan *et al.* have shown that immediate post-extraction bleeding in healthy subjects subsides within a maximum of 15 min after tooth extraction [7]. After subsidence of this relatively intense immediate post-extraction bleeding, the wound may continue to release small amounts of blood for up to 12 h [8]. These small amounts of blood are mixed with a larger amount of saliva (mere blood-tinged saliva) and can make the situation look a lot more dramatic to the uninformed patient than it really is. Such mild oozing sounds reasonable, considering that post-extraction socket is an open intraoral wound left to heal by secondary intention, being vulnerable to mechanical or thermal disturbance during mastication of food. Moreover, it has been claimed that the fibrinolytic activity of the saliva may also contribute to persistent oozing of intraoral wounds [9]. This situation should be considered as a physiological phenomenon and it should not be mistaken as recurrent haemorrhage.

Clinically overt sign of recurrent haemorrhage should be considered as the presence of aggressive oozing haemorrhage that continuously fills the oral cavity or the formation of “liver clot” or “currant jelly clot”, which is a dark red, jelly-like mass that forms over the tooth socket without actually stopping the bleeding Fig. (**1**). This blood clot mass tends to increase gradually in size as the socket continues to ooze blood underneath it. If the “liver clot” is ruptured, the socket restarts aggressively oozing blood until a new one is formed, and so on. Repetition of this situation and ingestion of large amounts of blood or clots may cause nausea and vomiting.

Immediate post-extraction bleeding and consequential oozing may be prolonged by both local and systemic factors. Local factors include wound site extent (damage caused by elevators and forceps or number of extracted teeth) and presence of inflammation (gingival or periodontal disease). Systemic factors include coagulation disorders or anticoagulant medication.

A critical question about immediate post-extraction bleeding and consequential oozing is the total amount of blood that could be lost. According to the American College of Surgeons Advanced Trauma Life Support (ATLS) classification, a blood loss <750 ml (15% of total blood volume) is unlikely to cause any significant haemodynamic compromise [10]. Although quantifying blood loss in oral surgery remains a rigorous goal in everyday clinical practice, studies demonstrated that in patients with normal haemostasis undergoing dental extractions blood loss rarely exceeds 750 ml, even in more complex interventions (*e.g.* multiple extractions with alveoloplasty) [11-27]. Usually, a simple non-surgical extraction of a single tooth does not seem to cause more than 5-10 ml of blood loss. Prolonged surgery time and periodontal inflammation seem to increase blood loss, while use of a local vasoconstrictor reduces blood loss to the half [11, 13, 17-20].

The severity of exodontia haemorrhage has been arbitrarily defined by several authors either by quantitative or qualitative criteria (Table **1**) [8, 28-32].

We have to bear in mind that the 750 ml blood loss mentioned in the ATLS classification refers to an acute setting with overt and rapid bleeding [10]. This leads to hypovolemia and decreased oxygen delivery to the tissues, initiating a cascade of stress responses to preserve adequate blood flow to vital organs [33]. On the other hand, post-extraction bleeding, as mentioned above, is usually slow and occult with small amounts of blood released from the wound for several hours.

The question remains whether post-exodontia bleeding, although not capable of leading to acute hypovolemia with adrenergic stimulation, could lead to anaemia if continued long-term. Such a type of slow bleeding could, in theory, lead to iron deficiency anaemia (in several weeks or months) and decreased oxygen delivery to the tissues if large amounts of blood are lost. Nevertheless, it is extremely improbable to encounter symptoms of severe anaemia after a simple dental extraction: Post-extraction bleeding is of capillary origin, with slow flow and small amounts of blood, the feeling of blood in the mouth immediately alerts the patient, the bleeding site is usually obvious to both the patient and the doctor and accessible for a timely and appropriate management [33, 34].

Other haemorrhagic complications may include intraoral or facial ecchymoses and haematomas of head and neck spaces. Such complications are associated rather with surgical or laborious dental extractions than with simple non-surgical uncomplicated extractions and they are more possible to occur in older patients because of their decreased tissue tone and increased fragility of their blood vessels. Ecchymosis is a non-elevated blue or purplish patch formed due to diffuse leakage of blood submucosally or subcutaneously from injured capillaries or sometimes venules. On the other hand, haematoma is a localized blood collection in an organ, space or tissue, usually associated with injury of larger blood vessels, but it can also be formed by injured capillaries in patients with concomitant coagulation disorders or receiving antithrombotic medication. Formation of a head and neck haematoma associated with dental extraction is rare, but it is a life-threatening complication given the potential for critical airway compromise, especially by haematomas in the sublingual or submandibular spaces.

## 3 MANAGEMENT OF POST EXODONTIA BLEEDING

A standard dental extraction session in a primary dental care (outpatient) setting consists of administration of local anaesthesia, tooth removal with root elevators and/or dental forceps, wound debridement and bleeding control with pressure pack *i.e*. pressure is applied on to the wound by instructing the patient to bite firmly on a piece of gauze for 30-60 min. The session is completed when adequate bleeding control has been achieved and the patient leaves the dental setting with specific post-operative instructions (hold the gauze in place with firm pressure for 30-60 min, no mouth rinsing, liquid or soft cold diet for the first 24 h, avoid smoking *etc*). It is also very important that all patients should be informed about mild oozing that may be experienced during the first post-operative hours and that this is an anticipated phenomenon for which they have nothing to worry about. Patients should comprehensively be informed about which situations could be considered alarming, such as recurrent aggressive oozing, formation of “liver clot”, neck swelling or dyscataposia (difficulty in swallowing) [2-4].

**Fig. (2):**
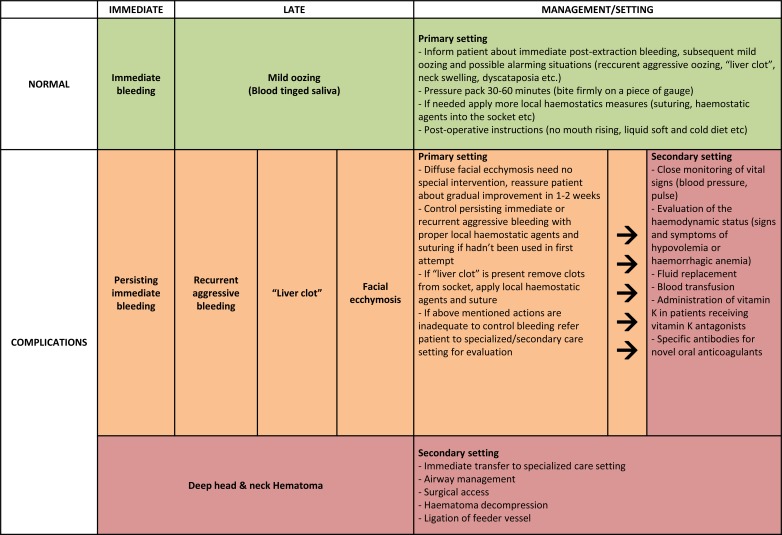
Post-extraction bleeding types, complications and management (Green: Minor, Yellow: Clinically Significant, Red: Life-Threatening).

A summarized approach to the management of post-exodontia bleeding according to its type, time of onset, complications and severity is provided in (Fig. **2**).

## 4 BLEEDING DEFINITIONS

Given the frequency of dental extractions in the aged population it is reasonable to assume that a large proportion of patients included in clinical studies will probably need a dental extraction. Thus, the need to develop a unified classification of post-exodontia bleeding is needed, in order to correctly define and rank its severity and determine therapeutic interventions and outcomes in cardiovascular antithrombotic clinical trials.

During the last 50 years, studies in oral surgery classify post-exodontia bleeding arbitrarily, either by means of the amount of blood that is lost during the procedure or by the time needed to stop the bleeding (Table **1**). As easily noted, the majority of these classifications refer to the immediate post-extraction bleeding not taking into account the late bleeding complications that may occur. Additionally, the methods of quantifying blood loss (gravimentric, photometry, plasma radioactivity) are difficult to obtain, ambiguous and with low repeatability, while bleeding time is a somehow subjective point based on the observer’s judgment about the event [8, 28-32].

However, the main common problem is that in the majority, the clinical significance of post-exodontia bleeding is overestimated [8, 28-32]. Using the existing classifications to define severe post-exodontia bleeding in the context of a trial of antithrombotics, a blood loss of 50 ml after a tooth extraction, defined as severe bleeding in existing classifications, is equalized with a life-threatening haemorrhagic complication like intracranial bleeding. This insidiously increases the incidence of bleeding end-points without an actual clinical impact, misleading outcomes and disrupting the sensitive balance between thromboembolic events and haemorrhage that is the final objective of these studies.

Bleeding definitions in antithrombotic cardiovascular clinical trials have progressed since the Thrombolysis In Myocardial Infarction (TIMI) bleeding criteria were introduced 30 years ago [35, 36]. These, as well as the Global Use of Strategies to Open Occluded Arteries (GUSTO) definition of bleeding proposed 5 years later, were used in the majority of studies in order to determine outcomes and drug or procedure safety [37]. Several other bleeding classifications have also been proposed and used in the context of a certain clinical trial [38, 39].

TIMI bleeding criteria were developed in the thrombolytic era and are well-suited to characterize severe acute events. However, nomenclature issues (major, minor), pitfalls in application (haemoglobin drops without clinically overt signs as major bleeding events, as well as uncertainty on the timing of assessing haemoglobin values) and the 3 different types of death in relation to bleeding pose certain limitations in their use [38]. GUSTO bleeding criteria were also introduced to identify significant bleeding in the setting of thrombolysis and define bleeding based on clinical parameters. It differs from several other definitions in that it does not quantify haemoglobin changes or the amount of blood transfused. The lack of an objective standardized criterion poses an inherent limitation in the GUSTO definition, making challenging the adjudication by a clinical events committee and the consistency of outcomes across different geographic regions, where thresholds for intervention and transfusion may vary, depending on local patterns of clinical practice, imaging use, blood banking, *etc*. [38].

Finally, the above definitions fail to successfully categorize and triage mild to moderate haemorrhagic events, underrating their importance and diminishing their associated risks, creating an illusion that they do not require monitoring and/or changes in the treatment regimens [39]. Recent bleeding definitions in Acute Coronary Syndrome (ACS) and Percutaneous Coronary Intervention (PCI) trials tried to combine both laboratory and clinical parameters in order to reinforce the strengths and overcome the limitations of the TIMI and GUSTO definitions [38].

However, substantial heterogeneity among the many bleeding definitions, lack of standardization which complicates adjudication and interpretation of comparisons of different antithrombotic agents and confusion in nomenclature (serious, severe, catastrophic, major, life-threatening), obscured the ability of clinical trials to meaningfully define the balance between safety and efficacy in cardiovascular interventions. Thus, in 2010 the Bleeding Academic Research Consortium (BARC) developed a consensus universal definition of bleeding to overcome, as much as possible, all the aforementioned heterogeneity and limitations of the different arbitrary bleeding classifications [38].

The BARC definition for bleeding attempted to incorporate information about the cause, site and severity of bleeding, to correlate with prognosis, to be able to direct specific diagnostic and treatment protocols, to be practical and easy to use, to discern small variations between therapies but with clinically meaningful conclusions and to put aside subjective nomenclature by using an alphanumeric grading scale for the severity instead of single descriptions (minor, major *etc.*). It comprises an objective, hierarchically graded, consensus classification for bleeding and it discerns 6 types of bleeding (from Type 0: No bleeding to Type 5: Fatal bleeding), also including CABG-related bleeding as Type 4 and recommends defining the timing of events at least at 7 days, 30 days and/or at the end of the trial [38].

## 5 CLASSIFICATION PROPOSAL

The lack of a universally accepted standardized definition for post-exodontia bleeding complications has a major contribution to the arbitrary perioperative management of antithrombotic medications in dental extractions. Dentists tend to ask for physician consultation before treating patients on antithrombotic medication. In most cases, physicians are not familiar with dental procedures and their associated bleeding risks, and they readily advise the discontinuation of antithrombotics perioperatively based on their knowledge about bleeding related to general or orthopaedic surgery. Moreover, dentists find the physician advice for antithrombotic interruption convenient, because it improves visibility of the surgical field and reduces the probability of facing a postoperative bleeding complication and its possible legal implications. However, optimal treatment planning for dental patients on antithrombotic therapy requires careful balancing of risks of bleeding and thrombosis. It is therefore important for both dental and medical practitioners to have a comprehensive knowledge of bleeding complications related to dental extractions and a mutual perspective when dealing with the management of dental patients on antithrombotic medication.

In order to overcome heterogeneity and limitations of the definitions for dental bleeding, we tried to develop a universal definition of post-exodontia bleeding, in accordance with the BARC bleeding definition that provides a trustworthy, well-constructed and oriented unified proposal Table (**2**).

## CONCLUSION

Post-exodontia bleeding, although usually not haemodynamically significant is always a worrying sign for the patient and is frequently encountered in every day clinical practice, given the high frequency of dental extractions. Many of these patients receive concomitant antithrombotic regimens, increasing the possibility for bleeding complications. The absence of a unified definition for post-exodontia bleeding creates a blurred setting in evaluating their clinical importance and making clear conclusions in antithrombotic cardiovascular trials. To fulfil this need, we propose a detailed classification of post-exodontia bleeding complications, based on the well-recognized BARC bleeding definition, hoping to eliminate heterogeneity in this field.

## Figures and Tables

**Fig. (1):**
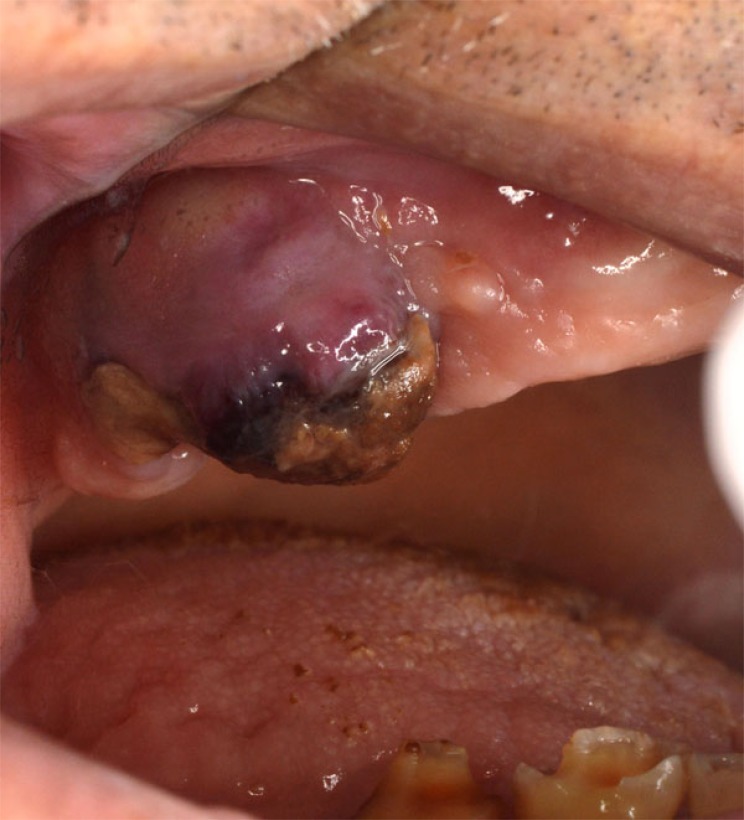
Liver clot” covering a post-extraction socket.

**Table 1:** Some classifications proposed for post-exodontia bleeding severity in patients on antithrombotic medication.

**STUDY**	**POST-EXODONTIA BLEEDING DEFINITION**
Ardekian *et al.*> 2000	Mild: Blood loss <20 ml Moderate: Blood loss 20-50 ml Severe: Blood loss >50 ml
Madan *et al.*> 2005	Severe: Blood loss >30 ml
Vicente-Barrero *et al.*> 2002	Mild: Bleeding time <5 min Moderate: Bleeding time ≥5 min Severe: requiring transfusion
Krishnan *et al.*> 2008	Severe: Bleeding 30 min after pressure pack applied
Morimoto *et al.*> 2008	Severe: Bleeding 30 min after pressure pack applied
Nooh 2009	Severe: Bleeding 30 min after pressure pack applied
Cardona-Tortajada *et al.*> 2009	Severe: Bleeding 10 min after pressure pack applied
Al-Belasy *et al.*> 2003	Mild: Bleeding time <20 min after pressure pack applied Moderate: Bleeding time ≥20 min after pressure pack applied with additional use of local haemostatic agents Severe: requiring suturing, vitamin K administration or transfusion
Lockhart *et al.*> 2003	Clinically significant: 1. Continues beyond 12 h; 2. Causes the patient to call or return to the dental practitioner or to the accident and emergency department; 3. Results in the development of a large haematoma or ecchymosis within the oral soft tissues; or 4. Requires a blood transfusion.

**Table 2:** Classification proposal for post-exodontia bleeding.

• **No bleeding** (corresponding to **Type 0 BARC**) • **Minor** (corresponding to **Type 1 BARC**) - Immediate post-extraction bleeding from socket which can be controlled on a first attempt with pressure pack and/or with other local haemostatic measures that are widely available in primary dental care settings (suturing, oxidized cellulose, gelatin or collagen sponge etc) or - Mild oozing from post-extraction socket (blood-tinged saliva) after patient leaving the dental setting which does not need special intervention to be controlled. Examples may include self-treating of oozing by pressing down with gauze for several minutes or cases of a patient seeking dental/medical attention but no special intervention is needed other than reassurance. • **Clinically significant** (corresponding to **Type 2 BARC**) - Persisting immediate post-extraction bleeding from socket which cannot be controlled on a first attempt with local haemostatic measures in primary dental setting, prompting evaluation or management in secondary or specialized healthcare setting but it does not fit the criteria for life-threatening bleeding or - Any clinically overt sign of recurrent aggressive post-extraction bleeding, formation of “liver clot”, large facial ecchymosis or persisting aggressive oozing continuing for more than 12 h requiring intervention by a healthcare professional to be controlled but does not fit the criteria for life-threatening bleeding • **Life-threatening** (corresponding to **Type 3 BARC**) Any immediate or late post-extraction bleeding complication with clinical, laboratory or/and imaging findings with specific healthcare provider responses: - Overt bleeding plus haemoglobin drop of 3 to < 5 g/dL* (provided haemoglobin drop is related to bleed) or any transfusion with overt bleeding (corresponding to **Type 3a BARC**) - Overt bleeding plus haemoglobin drop ≥ 5 g/dL* (provided haemoglobin drop is related to bleed) or bleeding requiring intravenous vasoactive agents or bleeding requiring surgical intervention for control (e.g. surgical evacuation of hematomas of deep head and neck spaces that may compromise airway, ligation or embolization of vessels to control bleeding) (corresponding to **Type 3b BARC**) • **Fatal** (corresponding to **Type 5 BARC**) - Probable fatal bleeding; no autopsy or imaging confirmation but clinically suspicious (corresponding to **Type 5a BARC**) - Definite fatal bleeding; overt bleeding or autopsy or imaging confirmation (corresponding to **Type 5b BARC**)
*Corrected for transfusion (1 U packed red blood cells or 1 U whole blood expected to increase haemoglobin by 1 g/dL). BARC: Bleeding Academic Research Consortium^38^
